# Melatonin Enhances the Mitochondrial Functionality of Brown Adipose Tissue in Obese—Diabetic Rats

**DOI:** 10.3390/antiox10091482

**Published:** 2021-09-17

**Authors:** Ahmad Agil, Miguel Navarro-Alarcon, Fatma Abo Zakaib Ali, Ashraf Albrakati, Diego Salagre, Cristina Campoy, Ehab Kotb Elmahallawy

**Affiliations:** 1 Department of Pharmacology and Neurosciences Institute, School of Medicine, University of Granada, 18016 Granada, Spain; 2 Biosanitary Research Institute of Granada (ibs.GRANADA), University Hospital of Granada, 18016 Granada, Spain; ccampoy@ugr.es; 3 Department of Nutrition and Bromatology, School of Pharmacy, University of Granada, 18071 Granada, Spain; nalarcon@ugr.es; 4 Department of Pathology and Clinical Pathology, Faculty of Veterinary Medicine, Sohag University, Sohag 82524, Egypt; fatma_ali@vet.sohag.edu.eg; 5 Department of Human Anatomy, College of Medicine, Taif University, P.O. Box 11099, Taif 21944, Saudi Arabia; a.albrakati@tu.edu.sa; 6 Department of Pediatric, School of Medicine, University of Granada, 18071 Granada, Spain; 7 Department of Zoonoses, Faculty of Veterinary Medicine, Sohag University, Sohag 82524, Egypt; eehaa@unileon.es

**Keywords:** melatonin, brown adipose tissue, mitochondrial function, Zücker diabetic fatty rat

## Abstract

Developing novel drugs/targets remains a major effort toward controlling obesity-related type 2 diabetes (diabesity). Melatonin controls obesity and improves glucose homeostasis in rodents, mainly via the thermogenic effects of increasing the amount of brown adipose tissue (BAT) and increases in mitochondrial mass, amount of UCP1 protein, and thermogenic capacity. Importantly, mitochondria are widely known as a therapeutic target of melatonin; however, direct evidence of melatonin on the function of mitochondria from BAT and the mechanistic pathways underlying these effects remains lacking. This study investigated the effects of melatonin on mitochondrial functions in BAT of Zücker diabetic fatty (ZDF) rats, which are considered a model of obesity-related type 2 diabetes mellitus (T2DM). At five weeks of age, Zücker lean (ZL) and ZDF rats were subdivided into two groups, consisting of control and treated with oral melatonin for six weeks. Mitochondria were isolated from BAT of animals from both groups, using subcellular fractionation techniques, followed by measurement of several mitochondrial parameters, including respiratory control ratio (RCR), phosphorylation coefficient (ADP/O ratio), ATP production, level of mitochondrial nitrites, superoxide dismutase activity, and alteration in the mitochondrial permeability transition pore (mPTP). Interestingly, melatonin increased RCR in mitochondria from brown fat of both ZL and ZDF rats through the reduction of the proton leak component of respiration (state 4). In addition, melatonin improved the ADP/O ratio in obese rats and augmented ATP production in lean rats. Further, melatonin reduced mitochondrial nitrosative and oxidative status by decreasing nitrite levels and increasing superoxide dismutase activity in both groups, as well as inhibited mPTP in mitochondria isolated from brown fat. Taken together, the present data revealed that chronic oral administration of melatonin improved mitochondrial respiration in brown adipocytes, while decreasing oxidative and nitrosative stress and susceptibility of adipocytes to apoptosis in ZDF rats, suggesting a beneficial use in the treatment of diabesity. Further research regarding the molecular mechanisms underlying the effects of melatonin on diabesity is warranted.

## 1. Introduction

Mitochondrial dysfunction is a main mechanism underlying insulin resistance in obesity and type 2 diabetes mellitus (T2DM) [1,2]. An imbalance between energy intake and expenditure in tissues related to the regulation of whole-body energy homeostasis, including liver, muscle, white, beige, and brown adipose tissues (BAT), results in impairment of mitochondrial function [3,4]. Taken into consideration, mitochondrial dysfunction encompasses a broad range of abnormalities, but primarily a reduced ratio of ATP production from cellular respiration [5]. In addition, alterations in other processes, such as the generation and detoxification of reactive oxygen/nitrogen species (ROS/RNS), organelle biogenesis, and the regulation of mitochondrial matrix calcium are involved [5].

Of importance, increased intramitochondrial oxidative and nitrosative stress and elevated cytosolic calcium concentration, along with increased mitochondrial permeability, contribute to various types of mitochondrial dysfunction [6,7,8]. The neurohormone melatonin is mainly produced by the pineal gland during darkness [9], in addition to many other tissues [10], where it mediates photoperiodic entrainment of endogenous circadian rhythms [11,12]. Additionally, melatonin is a potent antioxidant [13,14,15,16,17] that participates in energy homeostasis [17,18,19] and confers potential benefits in several physiological and pathological conditions [20,21,22].

Importantly, several studies have demonstrated that melatonin supplementation limited obesity in rodents without affecting food intake or locomotor activity [23,24,25,26,27]. In previous studies, we demonstrated that chronic oral administration of melatonin induced browning of inguinal white adipose tissue [4] and increased the mass and activity of BAT, which together have thermogenic properties [28].

Furthermore, previous reports have documented that melatonin enhances mitochondrial respiration and alleviates oxidative stress in different pathological conditions and in a broad variety of organs and tissues [14,29], including the brain [30,31] and liver [32,33,34,35,36], as well as cardiac [37,38,39] and skeletal muscle [40]. Additionally, a body of experimental evidence suggests that melatonin directly inhibits the mitochondrial permeability transition pore (mPTP) [41], which is involved in signaling apoptotic and necrotic cell death [42]. Data from our laboratory showed that melatonin improved mitochondrial function of different organs and tissues like liver, kidney, and inguinal white adipose tissue in diabesity conditions in Zücker diabetic fatty (ZDF) animals [20,36,43].

However, no available data were reported on the potential influence of melatonin on the mitochondrial function of BAT in obesity or diabetes. Thus, the present work investigated the effect of chronic oral melatonin administration on the mitochondrial function of BAT isolated from obese–diabetic ZDF rats (a well-established model of metabolic syndrome and T2DM) and their Zücker lean (ZL) littermates.

## 2. Materials and Methods

### 2.1. Ethical Statement

Ethical approval was obtained as described by the ethical standards of the University of Granada (Granada, Spain), which is in accordance with the European Union guidelines for animal care and protection. The ethical approval number of the project is 4-09-2016-CEEA.

### 2.2. Reagents

All reagents, chemicals, and materials used in the present study were of analytical grade and the highest purity. Melatonin and other chemicals were purchased from Sigma Chemicals (Madrid, Spain).

### 2.3. Animals and Experimental Protocols

In this work, male ZDF rats (fa/fa *n* = 16) and male lean littermates (ZL, fa/- *n* = 16) were obtained at the age of 5 weeks from Charles River laboratory (Barcelona, Spain). Animals were then maintained on Purina 5008 rat chow (carbohydrates 58.5%, protein 23%, ash 8%, fat 6.5%, and fiber 4%), and housed at rate of two rats per clear plastic cage in a climate-controlled room. Animals were maintained at 28–30 °C and 30–40% relative humidity, with a 12 h dark/light cycle. The animals were acclimated for one week after their arrival to room conditions combined with recording water intake. The experimental animals (ZL and ZDF rats) were then subdivided into two groups: (1) the first group included animals treated with melatonin in their drinking water for 6 weeks and comprised the two melatonin-treated subgroups M-ZDF (N = 8 rats) and M-ZL (N = 8 rats); and (2) vehicle-treated control subgroups, which included C-ZDF (N = 8 rats) and C-ZL (N = 8 rats). Melatonin was dissolved in absolute ethanol with a final concentration of 0.066% (*w*/*v*) ethanol and diluted in the drinking water to yield a dose of 10 mg/kg body weight (BW)/day. Water intake and BW were recorded twice weekly during the experiments. Importantly, melatonin and vehicle solutions were freshly prepared twice a week, the drinking fluid was changed twice weekly, and the dose of melatonin was adjusted according the BW of each animal throughout the study period. Aluminum foil was used to cover the bottles of drinking water to protect the mixture from light.

#### 2.3.1. Mitochondrial Preparation

Animals used in the experiments were anesthetized using sodium thiobarbital (thiopental) and sacrificed at the end of the treatment period. BAT samples (approximately 300 mg) were excised from brown regions of the interscapular fat pad. Adipocyte mitochondria were then isolated from these depots using a serial centrifugation method according to a previously described protocol with slight modifications [44]. Tissues were removed, excised, washed with cold saline, and homogenized in isolation medium containing 10 mm Tris, 250 mm sucrose, 0.5 mm Na_2_EDTA, and 1 g/L free fatty acid bovine serum albumin (BSA) (pH 7.4, 4 °C) with a Teflon pestle. The homogenate was then centrifuged at 1000× *g* for 10 min at 4 °C, followed by centrifugation of the supernatant at 15,000× *g* for 20 min at 4 °C. The resultant pellet was then resuspended in 1 mL of isolation medium without BSA, and an aliquot was frozen for measurement of protein concentration. The remaining mitochondrial suspensions were then centrifuged at 15,000× *g* for 20 min at 4 °C, followed by resuspension in 1 mL of respiration buffer (200 mm sucrose, 20 mm HEPES, 20 mm taurine, 10 mm KH_2_PO_4_, 3 mm MgCl_2_, 0.5 mm EGTA, and 1 g/L free fatty acid BSA), followed by keeping the mitochondrial suspensions on ice for 10–15 min.

#### 2.3.2. Mitochondrial High-Resolution Respirometry

Mitochondrial respiration was investigated using a high-resolution Oxygraph-2k (Oroboros Instruments, Innsbruck, Austria), which was composed of a two-chamber respirometer with a Peltier thermostat and electromagnetic stirrers [45]. The analysis was conducted in 2 mL of respiration medium that was previously equilibrated in each chamber with air at 30 °C and then stirred at 750 rpm until a stable air saturation signal was reached. All experiments were performed using a final concentration of 0.2–0.3 mg/mL fresh protein of isolated mitochondria in respiratory buffer. The isolated mitochondria were suspended in respiration buffer supplemented with 5 mM glutamate and 2.5 mM malate or with 5 mm succinate in the presence of rotenone as an energizing substrate. This was followed by measurement and recording of oxygen flux (J_O2_) at 30 °C in a constantly stirred oxygraph vessel after the successive addition of 1 mm ADP (state 3_ADP_ or OXPHOS capacity) with 0.75 mm oligomycin, which acted as an ATPase inhibitor (state 4 or leak respiration). Expression of results was as pmol of oxygen consumed/min mg protein per respiratory state. Measurements were taken at 0.2 s intervals for 15–20 min, and a computer-driven data acquisition system (DatLab, Innsbruck, Austria) was used for recording these measurements. The respiratory control ratio (RCR), determined as the ratio of state 3 to state 4, was used as a general measure of mitochondrial function [5]. Determination of the phosphorylation coefficient (ADP/O ratio) was also performed and defined as the amount of ADP added to the respiratory medium divided by nanograms of oxygen consumed during state 3.

#### 2.3.3. ATP Concentration

Quantification of the concentration of the ATP in mitochondrial aliquots was performed by bioluminescence [37] using a commercial kit (Molecular Probes, A22066; Invitrogen, Madrid, Spain) following the manufacturer’s instructions. The minimum detectable amount of ATP was 0.1 pM/mg protein. The results of ATP were quantified as micrograms of ATP per milligram of depot wet weight and expressed as nmol/mg protein. Protein concentration in aliquots was adjusted to 0.5 mg/mL protein concentration.

#### 2.3.4. Determination of Mitochondrial Nitrites

Mitochondrial nitrites were detected spectrophotometrically by a Griess diazotization reaction [46] using a Griess Reagent Kit (G-7921; Molecular Probes, Life Technologies, Madrid, Spain), according to the instructions of the manufacturer. The optimum wavelength for measurement was 548 nm.

#### 2.3.5. Measurement of Superoxide Dismutase (SOD) Mitochondrial Activity

SOD (Mn and Cu/Zn) activity of isolated mitochondria was measured using an SOD Assay Kit (19160; Sigma-Aldrich, Buchs, Switzerland), following the kit’s instructions. The results (SOD activity) were then expressed as a percentage of the inhibition rate (%), and its minimum detectable amount was 0.001 U/mL.

#### 2.3.6. Measurement of Calcium Retention Capacity and mPTP Activity

The sensitivity of mPTP opening to calcium ions and the calcium retention capacity of isolated mitochondria were assessed in the presence of 0.25 μM Oregon green using a fluorometric assay. Each well of a 96-well plate was filled with 0.5 mg/mL of mitochondrial solution, 10 μL of a respiratory substrate, and 0.25 μM of the fluorochrome Oregon green (Invitrogen, Madrid, Spain) in a final volume of 100 μL. The baseline fluorescence was then measured firstly without addition of calcium. An average of four pulses of 0.50 μm CaCl_2_ were then added, which produced fluorescence in the presence of Oregon green. Briefly, calcium was then retained and accumulated in mitochondria in the presence of ATP. The retained mitochondrial calcium resulted in an overload and induced opening of mPTP, resulting in the release of a large quantity of calcium that could be then quantitated using a fluorometer. Likewise, 1 μm cyclosporine A (CsA) (Sigma-Aldrich, Madrid, Spain) was used as the standard mPTP inhibitor [47]. The experimental groups were as follows: (1) control (mitochondria from BAT of white and beige fat depots of untreated C-ZL and C-ZDF rats) challenged with 250 µM Ca^2+^ overload alone; (2) melatonin (mitochondria isolated from BAT of white and beige fat depots of M-ZL and M-ZDF rats); (3) CsA (mitochondria from BAT of white and beige fat depots of C-ZL and C-ZDF rats) was challenged with calcium in the presence of CsA; and (4) melatonin + CsA (mitochondria isolated from white and beige fat depots of melatonin-treated ZL and ZDF rats) challenged with calcium in the presence of CsA. mPTP opening was measured from signals over time and expressed as the area under the curve (AUC), and four replicates were made for each sample.

### 2.4. Statistical Analysis

Data were expressed as mean ± SD and compared among groups using two-way analysis of variance (ANOVA) followed by the Tukey post hoc test. SPSS for Windows version 15.0 (SPSS Inc., Michigan, IL, USA) was performed for analyses of the data. *p <* 0.05 was considered statistically significant.

## 3. Results

### 3.1. The Effect of Melatonin Treatment on Mitochondrial Respiratory (States) in BAT-Isolated Mitochondria

**Figure 1:**
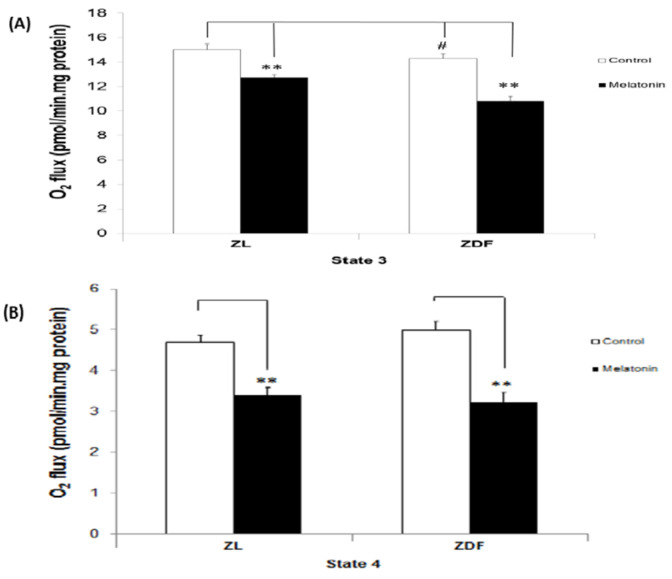
The effect of melatonin treatment on respiratory states in isolated mitochondria from brown fat depots of control (untreated) and melatonin-treated ZDF and ZL rats. (**A**) The upper panel shows state 3, representing oxygen flux, while producing ATP in response to ADP pulses in the presence of substrates. (**B**) The lower panel illustrates state 4, or leak respiration, which shows oxygen flux in the absence of ATP synthesis. Glutamate/malate was used as the respiratory substrates. C, control; M, melatonin; ZL, Zücker lean rats; ZDF, Zücker diabetic fatty rats. Values are shown as mean ± SD. Superscript characters show significant differences determined by two-way analysis of variance (ANOVA) followed by the Tukey post hoc test (** p < 0.01, M-ZDF compared with C-ZDF rats; ^#^ *p* < 0.05, C-ZDF compared with C-ZL rats).

Figure 1 depicts the rate of oxygen uptake proportional to oxygen flux (expressed as pmol/min mg protein), which rose sharply after ADP addition, followed by a rapid reduction due to ADP depletion, as it was phosphorylated to ATP (state 3). Accordingly, state 3 represented the oxygen flux while producing ATP in response to ADP pulses in the presence of substrates and BAT-isolated mitochondria. As shown in Figure 1A, BAT mitochondria isolated from both melatonin-treated ZL and ZDF animals had significantly reduced oxygen flux in state 3 compared with untreated controls in both brown fat depots of ZL and ZDF phenotypes (15.02 ± 0. 46 versus 12.74 ± 0.22 pmol/min mg protein in C-ZL and M-ZL rats, respectively, *p* < 0.05; and 14.30 ± 0.37 versus 10.81 ± 0.34 pmol/min mg protein in C-ZDF and M-ZDF rats, respectively, *p* < 0.05). In contrast, a small but significant decrease in the value of state 3 of obese–diabetic compared to lean animals (controls) was observed. In accordance with state 4, which represented the oxygen flux in the absence of ATP synthesis in mitochondria isolated from brown fat of both ZL and ZDF melatonin-treated animals, it was reduced in this work (Figure 1B). The leak respiration was 28% lower in ZL (oxygen flux of 4.70 ± 0.16 versus 3.38 ± 0.20 pmol/min mg protein in C-ZL and M-ZL, respectively, *p* < 0.05) and 36% lower in ZDF (oxygen flux of 5.00 ± 0.20 versus 3.22 ± 0.23 pmol/min mg protein in C-ZDF and M-ZDF, respectively, *p* < 0.05) rats. However, no statistical differences were found in the values of state 4 for the two controls and obese–diabetics compared with lean animals (C-ZDF versus C-ZL and M-ZDF versus M-ZL, respectively).

### 3.2. The Effect of Melatonin Treatment on RCR of Mitochondria from BAT

**Figure 2:**
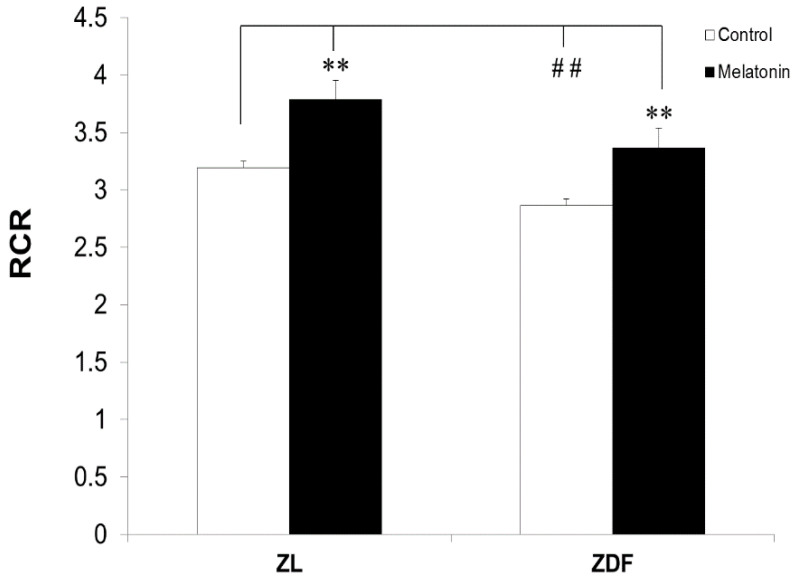
Respiratory control ratio (RCR) of mitochondria isolated from brown adipose fat depots of control (untreated) and melatonin-treated ZDF and ZL rats. RCR was defined as the ratio of state 3 to state 4. Data are shown as mean ± SD. ZL, Zücker lean rats; ZDF, Zücker diabetic fatty rats. Superscript characters indicate significant differences determined by two-way ANOVA followed by the Tukey post hoc test (** p < 0.01, M-ZDF compared with C-ZDF and M-ZL compared with C-ZL animals; ^##^ *p* < 0.01, C-ZDF and M-ZDF compared with C-ZL rats).

Next, we found that the RCR increased in BAT-isolated mitochondria of melatonin-treated ZL (from 3.19 ± 0.06 to 3.78 ± 0.17 μmol/mg protein, *p* < 0.01) and ZDF (from 2.86 ± 0.06 to 3.36 ± 0.17 μmol/mg protein, *p* < 0.01) rats compared to the respective controls (Figure 2). In contrast, the BAT RCR decreased in diabetic obese (C-ZDF) rats from 3.19 ± 0.03 μmol/mg protein in C-ZL to 2.86 ± 0.03 μmol/mg protein in C-ZDF animals (*p* < 0.05).

### 3.3. Melatonin Increased the Respiratory Phosphorylation Coefficient (ADP/O Ratio) of BAT-Isolated Mitochondria

**Figure 3:**
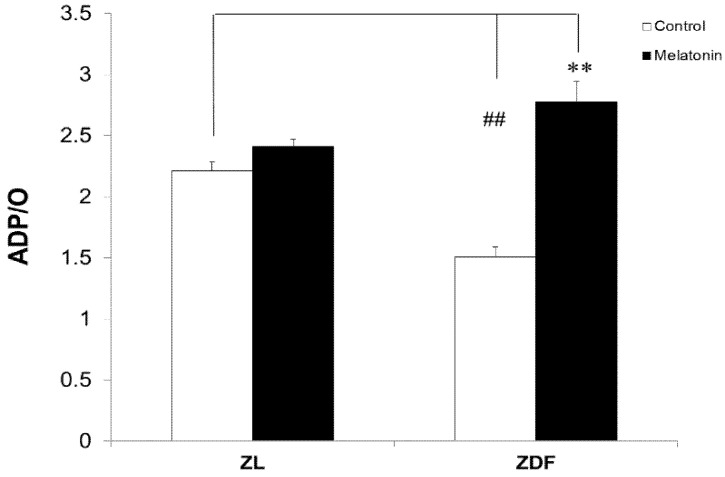
Respiratory phosphorylation coefficient (ADP/O ratio) of brown adipose tissue (BAT)-isolated mitochondria from control (untreated) and melatonin-treated ZDF and ZL rats. Values are shown as mean ± SD. Superscript characters show significant differences determined by two-way ANOVA followed by the Tukey post hoc test (** p < 0.01, M-ZDF compared with C-ZDF rats; ^##^ *p* < 0.01, C-ZDF and M-ZDF compared with C-ZL rats).

As depicted in Figure 3, mitochondrial BAT of M-ZDF rats showed a significant increase in phosphorylation efficiency (ADP/O ratio) compared with the C-ZDF group (*p* < 0.01). Furthermore, the positive effects of melatonin on diabetic fatty rats not only resulted in the complete restoration of phosphorylation efficiency, but also raised its values above normal mitochondrial respiration parameters versus the C-ZL group. However, the phosphorylation efficiency of melatonin-treated lean rats was not statistically different compared to their controls.

### 3.4. Melatonin Nonsignificantly Increased ATP Levels of Mitochondria from BAT

**Figure 4:**
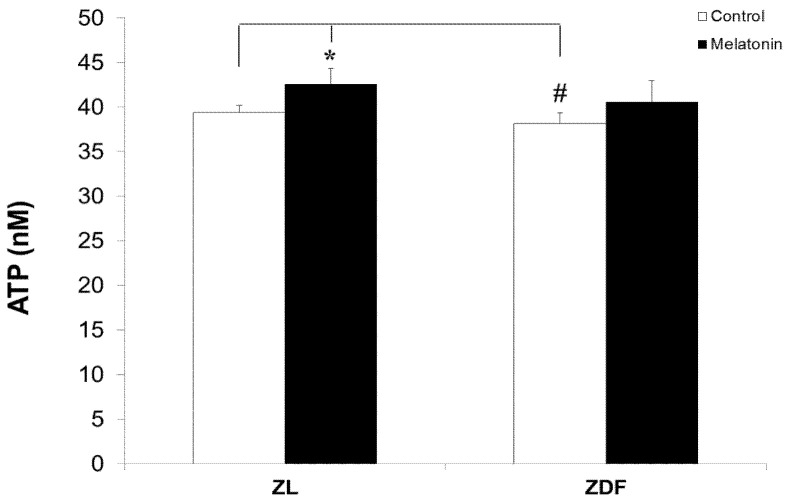
ATP levels of BAT-isolated mitochondria of different treated groups. Data were normalized for variations in mitochondrial content in an equivalent amount of tissue (micrograms of ATP/mg of tissue wet weight), adjusted to 0.5 mg/mL protein concentration, and expressed as nmol/mg protein. Data are shown as mean ± SD. Superscript characters show significant differences determined by two-way ANOVA followed by the Tukey post hoc test (* p < 0.01, M-ZL compared with C-ZL rats; ^#^ *p* < 0.05, C-ZDF compared with C-ZL rats).

We found no significant differences in the ATP content in response to melatonin treatment in obese rats; however, ZL rats experienced a small but significant elevation in their ATP levels after treatment with melatonin (Figure 4). As depicted in Figure 4, the ATP levels in brown fat depots were higher in C-ZL rats (39.42 ± 0.78 nM) versus those of diabetic obese (C-ZDF) rats (38.14 ± 1.20 nM). On the other hand, animals treated with melatonin had a small but significantly higher level of ATP (42.54 ± 1.82 nM p in M-ZL versus 39.42 nμ in C-ZL, *p* < 0.05).

### 3.5. Melatonin Reduced Levels of Mitochondrial Nitrites from Brown Fat Depots

**Figure 5:**
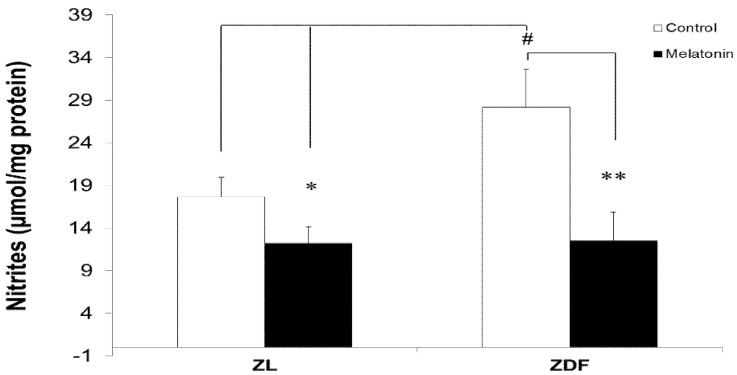
The effect of melatonin on nitrite levels in mitochondria isolated from BAT fat depots of control (untreated) and melatonin-treated ZDF and ZL rats. Values are shown as mean ± SD. Superscript letters express significant difference measured using two-way ANOVA followed by the Tukey post hoc test. * p < 0.05 (M-ZL compared with C-ZL rats), ** p < 0.01 (M-ZDF compared with C-ZDF rats), and ^#^ *p* < 0.05 (C-ZDF and M-ZDF compared with C-ZL rats).

Next, we evaluated the effect of melatonin treatment on BAT mitochondrial nitrite levels and found that the levels of mitochondrial nitrites in brown fat depots were higher in diabetic obese (C-ZDF) rats (28.18 ± 2.47 μmol/mg protein) versus those of C-ZL (17.67 ± 2.28 μmol/mg protein; *p* < 0.05) rats (Figure 5). However, animals treated with melatonin had lower but nonsignificant nitrite levels (20.7 ± 0.7 versus 28.18 ± 4.47 μmol/mg protein in C-ZL and C-ZDF, respectively). Notably, treatment with melatonin significantly reduced the levels of nitrites in BAT-isolated mitochondria of ZL (from 17.67 ± 2.28 to 12.20 ± 1.92 μmol/mg protein, *p* < 0.05) and ZDF (from 28.18 ± 2.47 to 12.51 ± 3.35 μmol/mg protein, *p* < 0.05) rats.

### 3.6. Melatonin Increased SOD Activity of BAT-Derived Mitochondria

**Figure 6:**
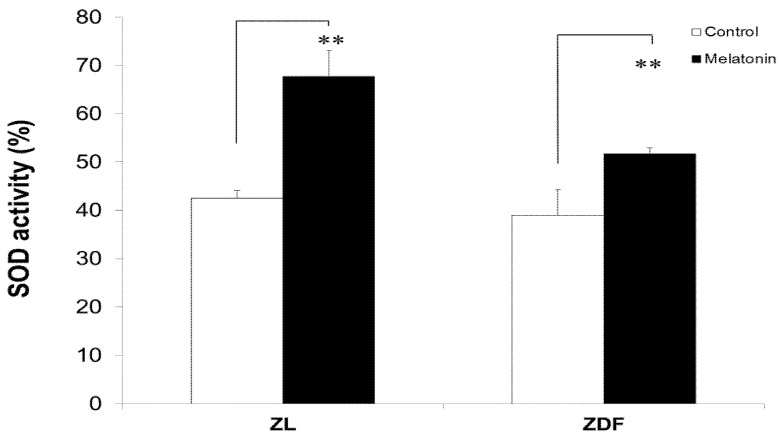
The effect of melatonin treatment on superoxide dismutase (SOD) activity in mitochondria isolated from BAT of control (untreated) and melatonin-treated ZDF and ZL rats. Values are expressed as mean ± SD. Superscript letters identify significant difference measured using two-way ANOVA followed by the Tukey post hoc test. ZL, Zücker lean rats; ZDF, Zücker diabetic fatty rats. ** p < 0.01, M-ZDF compared with C-ZDF rats and M-ZL compared with C-ZL animals.

It should be stressed that melatonin-treated lean animals had higher SOD activity in mitochondria isolated from brown fat depots compared to untreated controls, increasing from 42.51% ± 1.54% to 67.72% ± 5.38% (*p* < 0.05). Similarly, SOD activity increased in melatonin-treated ZDF animals from 38.94% ± 5.34% to 51.70% ± 1.28% (*p* < 0.05), as shown in Figure 6. In contrast, no statistically significant changes in SOD activity were observed in BAT-isolated mitochondria of either obese or lean control animals.

### 3.7. Melatonin Treatment Altered Calcium Retention Capacity and Sensitivity of mPTP Opening

**Figure 7:**
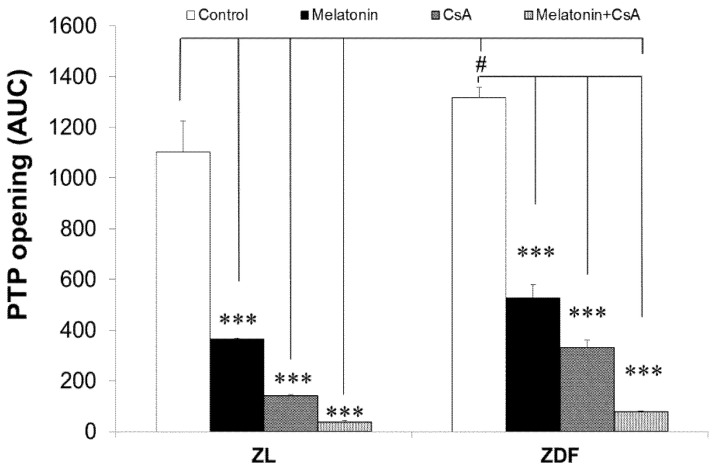
The effect of melatonin treatment on calcium-induced permeability transition pore (PTP) in mitochondria isolated from BAT of control (untreated) and melatonin-treated ZDF and ZL rats. The effect of cyclosporine A (CsA) was included as a classical inhibitor of mitochondrial PTP. Data are shown as mean ± SD of the area under the curve (AUC). Superscript letters identify significant difference measured using two-way ANOVA followed by the Tukey post hoc test. ZL, Zücker lean rats; ZDF, Zücker diabetic fatty rats. *** p < 0.001 and *# p* < 0.05.

As depicted in Figure 7, pretreatment with melatonin during the in vivo stage of experiments resulted in notorious inhibition of calcium (Ca^2+^)-induced mPTP opening in isolated mitochondria “in vitro” from brown fat in both lean (by 67%, *p* < 0.001) and obese (by 60%, *p* < 0.01) rats. However, CsA inhibited the activity of mPTP by 87% and 76% in BAT-isolated mitochondria of ZL and ZDF rats, respectively. Furthermore, CsA provoked significantly greater inhibition (97% and 94%) of mPTP in mitochondria from melatonin-treated ZL and ZDF animals, respectively. The addition of Ca^2+^ or melatonin in mitochondrial suspensions inhibited the release of accumulated Ca^2+^ in all isolated mitochondrial fractions, and this inhibition was potentiated by CsA. We found that the AUC was significantly lower for groups treated with melatonin than controls, indicating that the mPTP opens later with higher Ca^2+^.

## 4. Discussion

This study reported novel data on the potential benefits of oral melatonin supplementation in improved functionality of mitochondria isolated from BAT of obese–diabetic ZDF rats. The work also explored the possible underlying mechanistic pathways involved in improved mitochondrial respiration, decreased nitrite production, improved antioxidative capacity, and inhibition of mPTP. Previously, we showed that melatonin induced BAT mass and thermogenic activity in diabetic Zücker rats [28], and the observed increase in BAT mass and functionally after melatonin treatment was accompanied by increases in mitochondrial mass, citrate synthase activity, and complex I and IV. Furthermore, obese–diabetic ZDF rats treated with melatonin displayed marked thermogenic effects, characterized by a remarkable increase in protein expression of isolated BAT mitochondrial UCP1 and GPD binding and sensitized thermogenic response to cold exposure in vivo [28]. However, no information was available regarding the mechanistic pathways of this conversion or the potential influence of melatonin on BAT-derived mitochondrial functions.

Metabolic disorders of obesity and diabetes may be caused or exacerbated by adipocyte mitochondrial dysfunction, resulting in a series of events including less-effective substrate oxidation, increased oxido-nitrosative stress and calcium permeability, and apoptosis, all of which contribute to insulin resistance [48]. Clearly, the investigation of novel drug targets that counteract these actions may be helpful in combating diabesity and its metabolic complications, which are found worldwide.

Several previous reports have shown the promising effects of melatonin in improving mitochondrial efficiency in various pathological conditions resulting from a wide range of free scavenger, antioxidative, anti-inflammatory, and metabolic effects, such as improvement in BW gain and glucose hemostasis [21,26,49]. When taken into account, the RCR is considered the best measure of mitochondrial function because it encapsulates the principal function of ATP generation [5]. Our results showed that the mitochondria of brown adipocytes of obese–diabetic animals exhibited significantly lower ATP production versus that of lean littermates. Furthermore, treatment with melatonin significantly increased ATP production in BAT of lean (ZL) animals. As expected, BAT-derived mitochondria exhibited increased uncoupled respiration, which may be attributed to their thermogenic properties. The chronic administration of melatonin also significantly increased OXPHOS capacity of mitochondria isolated from brown adipocytes in obese–diabetic (and not in lean) animals, a finding that contributes to the thermogenic properties of melatonin in brown fat [4,28], and was in full agreement with our previous data showing that melatonin treatment of obese rats restored the amount UCP1 protein in the BAT depots of obese–diabetic and not lean rats [28]. Additionally, these findings aligned with those of previous reports on the same strain of rats [26,49], showing that melatonin treatment improved both the excess weight loss and long-term hyperglycemic glycated hemoglobin (HbA1c) concentrations in obese–diabetic animals (with damaged mitochondria), and was not able to reduce normal weight and HbA1C levels in lean rats (with healthy mitochondria). Thus, diabesity-related conditions may contribute to increased oxidative stress but decreased OXPHOS efficiency in BAT mitochondria of obese–diabetic rats through marked proton reduction. In this regard, a significant deterioration of brown mitochondrial respiration was observed in obese–diabetic C-ZDF rats compared with lean rats; a difference that was observed as a slight but significant reduction in state 3 and a decline in both ATP production and RCR with a reduction in oxidative phosphorylation efficiency (ADP/O), which was in line with our previous results in liver tissue of obese–diabetic fatty rats [36]. The decrease in phosphorylative system efficiency may be caused by a lack of adenine nucleotide translocator, an inner membrane protein that catalyzes the conversion of ADP to ATP. Furthermore, melatonin treatment increased the RCR of brown adipocyte mitochondria in both obese and lean rats, indicating that melatonin increased mitochondrial integrity by robustly increasing OXPHOS capacity, a point that was evidenced by a significant decrease in oxygen flux in state 3 of respiration and leak respiration in state 4 in both obese and lean phenotype animals. In addition, our results on mitochondrial bioenergetics agreed with those of other studies in the literature obtained on mitochondria from other tissues in different pathological conditions [50,51,52,53,54].

To date, few studies have described the effect of melatonin on mitochondrial function in obesity and diabetes. Zavodnik et al. [32] revealed that administration of melatonin in streptozotocin-diabetic rats rescued the RCE diminution in liver mitochondria isolated from these animals. Additionally, Cheshchevik et al. [50] reported that administration of melatonin corrected liver mitochondrial dysfunction in streptozotocin-induced diabetes.

RNS production increases in various pathological conditions such as diabetes and obesity, resulting in exacerbation of the mitochondrial damage manifested by increased nitroxidative lesion [55], as demonstrated in mitochondrial kidney cells in the same strain of ZDF animal model versus lean animals [20]. As expected, melatonin supplementation reduced the generation of nitrites and increased SOD activity in BAT-isolated mitochondria of the two rat phenotypes in the present work. The capacity of melatonin to reduce the oxidative and nitrosative status in BAT-derived mitochondria was in concordance with previous reports in several organs and tissues, including the brain, liver, and kidney [13,29,30,31,32,33,34,35,36]. Thus, we concluded that the beneficial effects of melatonin were related to improved mitochondrial function by counteracting mitochondrial oxidative and nitrosative stress, which also identified mitochondria as a therapeutic target for the antioxidant actions of melatonin.

Remarkably, one of the most striking effects of melatonin treatment in our study was the alteration of Ca^2+^-induced mPTP. The mPTP is the most prominent of all inner membrane channels and the gatekeeper of apoptotic and necrotic cell death [42,56,57,58]. Furthermore, mPTP is linked to various diseases [42,59,60]. The possible inhibitory effect of melatonin treatment on Ca^2+^-induced mPTP was studied and compared with that of the classical mPTP inhibitor CsA in several previous studies [47,61]. The activity of mPTP in our present work was induced by raising the Ca^2+^ concentration in the incubation media. Our study showed for the first time that the mPTP was altered in BAT-isolated mitochondria of obese–diabetic animals compared with lean ones. This finding was supported by increased opening of mPTP, whereas melatonin treatment caused a profound inhibition of Ca^2+^-induced opening of mPTP in BAT-isolated mitochondria in both lean and obese rats.

Similarly, an earlier report by our group concluded that melatonin treatment also markedly inhibited Ca^2+^-induced mPTP opening in mitochondria isolated from inguinal white fat in both lean and obese rats [28,43]. Importantly, melatonin has a notorious inhibitory action on mPTP that is comparable to the effect of CsA [47], and impedes the opening of mPTP by binding and inactivating cyclophilin D. In line with other studies, melatonin may protect mitochondria from mitochondria-induced cell death, which has been attributed to the higher affinity of melatonin to directly inhibit mPTP [41,62]. However, CsA exerted a greater inhibitory effect of mPTP in mitochondria from animals treated with melatonin versus untreated, suggesting a different mechanism of action for both inhibitors. It is worth pointing out that melatonin may also contribute to mPTP inhibition through its role in reducing mitochondrial oxido-nitrosative stress, which is considered an inducer of mPTP opening.

## 5. Conclusions

Taken together, our results demonstrated for the first time that melatonin treatment enhanced several mitochondrial functions of interscapular brown fat cells in obese–diabetic (ZDF) rats. The present findings, in relation to oxidative and nitrosative stress, calcium retention capacity, and oxygen consumption, contribute to a better understanding of the benefits of melatonin on diabesity. Further research is recommended to elucidate other potential mechanisms of melatonin in diabetes and examine the possible involvement of melatonin receptors in these actions.

## Data Availability

Data is contained within the article.
